# Highly specific Electrochemical Sensing of *Pseudomonas aeruginosa* in patients suffering from corneal ulcers: A comparative study

**DOI:** 10.1038/s41598-019-54667-0

**Published:** 2019-12-04

**Authors:** Marwa M. Khalifa, Amal A. Elkhawaga, Mona A. Hassan, Asmaa M. Zahran, Ahmed M. Fathalla, Waleed A. El-Said, Omnia El-Badawy

**Affiliations:** 1 Department of Microbiology and Immunology, Faculty of Pharmacy, Assiut University, Assiut, 71526 Egypt; 2 Department of Medical Microbiology and Immunology, Faculty of Medicine, Assiut University, Assiut, 71515 Egypt; 3 Department of Clinical Pathology, South Egypt Cancer Institute, Assiut University, Assiut, Egypt; 4 Department of Ophthalmology, Faculty of Medicine, Assiut University, Assiut, 71515 Egypt; 5 Department of Chemistry, Faculty of Science, Assiut University, Assiut, 71516 Egypt; 6 Chemistry Department, Faculty of Science, University of Jeddah, P.O. 80327, Jeddah, 21589 Saudi Arabia

**Keywords:** Biochemistry, Biotechnology, Microbiology

## Abstract

*Pseudomonas aeruginosa* is the most common pathogenic gram-negative bacteria causing corneal ulcers globally. In severe cases, often after trauma and eye injury, corneal destruction progresses rapidly and may be completed within 24–48 h causing blindness. In our preliminary work, we have established an ultrasensitive polyaniline (PANI)/gold nanoparticles (Au NPs)/indium tin oxide (ITO) modified sensor for rapid detection of pyocyanin (PYO) in *P*. *aeruginosa* infections with a linear range from 238 μM to 1.9 μM and a detection limit of 500 nM. In the present study, we evaluated the efficiency of the established modified electrochemical sensor in the diagnosis of *P*. *aeruginosa* in 50 samples collected from patients suffering from corneal ulcers. The obtained results were compared with the results gained by the screen-printed electrode, conventional techniques, automated identification method, and the amplification of the 16 s rRNA gene by PCR as a gold standard test for *P*. *aeruginosa* identification. We have found that the electrochemical detection of PYO by square wave voltammetry technique using PANI/Au NPs modified ITO electrode was the only technique showing 100% agreement with the molecular method in sensitivity, specificity, positive and negative predictive values when compared with the SPE, conventional and automated methods.

## Introduction

A bacterial corneal ulcer is an infection and inflammation of the cornea, producing impaired vision, light sensitivity, pain, and tears or discharge from the eye. Corneal destruction progresses rapidly and in severe cases, may be completed within 24–48 h, causing blindness, especially with highly virulent bacteria^1^. Predisposing factors for corneal ulcers include ocular surface diseases, ocular trauma, and the extensive use of contact lenses. Among a wide range of bacteria that cause corneal ulcers, *P*. *aeruginosa* is the most frequent and the most pathogenic. It can cause corneal perforation in less than 24 h from the onset of infection^2^. *P*. *aeruginosa* may acquire multidrug resistance, posing a significant challenge to its eradication with antimicrobial agents^3^. Hence, early identification of the bacterium is vital to avoid the establishment of chronic infection^4^.

Although using culture media for bacterial identification is a well-established technique in microbiology laboratories, it is not quantitative and takes 24 h or more to provide results^5^. Polymerase chain reaction (PCR) identification is nowadays available in an increasing number of laboratories. Still, PCR requires extensive sample preparation, uses expensive reagents, and is time-consuming^6^.

*P*. *aeruginosa* is known to produce a blue-green redox-active phenazine pigment known as pyocyanin (PYO) that can kill competing bacteria and mammalian cells by generating reactive oxygen intermediates^7^. *P*. *aeruginosa* produces large amounts of PYO during the early colonization phase^8^. It is exclusively produced by *P*. *aeruginosa*, making it potentially an excellent diagnostic biomarker^9^.

Electrochemical approaches are superior to the spectrophotometric methods of PYO detection in being more sensitive, cheap, fast, and direct methods of detecting PYO^5^. Hereafter, they became popular alternatives to the usual optical or separation based analytical techniques. Additionally, Alatraktchi and her collaborators have described an electrochemical method for selective identification and quantification of PYO^10^. The scanning electrochemical microscopy (SECM) was also used to observe PYO at the surface of biofilms with high resolution^11^. Voltammetric techniques were efficiently employed to detect and quantify PYO in human fluid samples^5,12,13^.

Gold nanostructures modified electrodes are now widely used as biosensors because of the exceptional electrical and optical characteristics and affinity to bind with biomolecules^14–24^. Likewise, the use of the Au modified ITO electrode has led to fast and ultrasensitive detection of some multidrug-resistant bacteria such as *E*. *coli* and *Staph*. *aureus*^20^. Likewise, polyaniline (PANI) is one of the promising conducting polymers^25^ that has gained much interest in recent electrochemical sensors researches^26,27^, with good conductivity, stability, and easy to prepare.

Hybridization of PANI with nanomaterials could endow great promise in the sensors field due to the enhancement of its electrical conductivity in addition to its capability to act as a scaffold for immobilization of the biological species^12,13^. However, none of the previously mentioned electroanalytical techniques for the PYO detection have employed PANI/Au NPs modified indium tin oxide (ITO) as an electrode-based electrochemical sensor.

Up to date, it is still challenging to discover novel and more sensitive approaches for rapid and accurate diagnosis of *P*. *aeruginosa* infections, which will aid in fast treatment decisions, particularly in cases of corneal ulcers. Recently, we have established a PANI/Au NPs/ITO modified electrode as a rapid ultra-sensitive technique for the detection of PYO in *P*. *aeruginosa* infections^28^. The PANI/Au NPs/ITO modified electrode with positive charges on its surface was selected to monitor PYO that is bearing a negative charge and hence could enhance the mass transfer rate based on the electrostatic attraction force.

The aim of the current study was to compare the results of the electrochemical biosensors using the established PANI/Au NPs/ITO modified electrode with the screen-printed electrode (SPE) and with the conventional, automated and molecular methods for *P*. *aeruginosa* detection in cases of corneal ulcers.

## Materials and Methods

Fifty patients with corneal ulcers were enrolled in this study. They were 27 females and 23 males. Their mean age was 50.5 ± 7 years. The study was conducted in the Medical Microbiology & Immunology Department, Faculty of Medicine, Assiut University, from November 2018 to February 2019. Electrochemical detection was done in the Chemistry department, Faculty of Science, Assiut University. Clinical diagnosis of corneal ulcers was performed in the Department of Ophthalmology, Assiut University Hospital using the fluorescein stain and slit-lamp examination. Patients who started antimicrobial therapy were excluded from this study.

### Sample collection

Corneal ulcer samples were collected from the patients by an ophthalmologist under complete aseptic conditions using a sterile cotton swab by swabbing the base of the corneal ulcer using gentle pressure with enough pressure to indent the cornea slightly. If the cornea was significantly thinned, less pressure was applied to avoid perforation^29^. The swab was placed in LB broth transport media (BIO BASIC INC, Canada) and was incubated at 37 °C for 24 h. LB is a rich medium, allowing rapid and high growth rates of different microorganisms and significantly higher concentrations of PYO were detected in the LB cultures^30^. The final pH of the LB broth culture at room temperature is found to be 7.4 (the physiological pH). Afterward, for the phenotypic identification of *P*. *aeruginosa*, all collected samples were subjected to cultures, biochemical tests, and automated system based identification. Electrochemical biosensors were used to measure PYO. Confirmation of diagnosis was done by the amplification of the 16 s rRNA gene of *P*. *aeruginosa*.

### Electrochemical identification of *P*. *aeruginosa* in corneal ulcer samples

The Autolab potentiostat instrument (Metrohm Model 663 VA, Netherlands) was used for all electrochemical experiments. It was connected to a three-electrode system; Nova software was used to control the system at room temperature. Two types of 3- electrode electrochemical cell systems were used for the electrochemical measurements:

- Voltammetric cell that consisted of a platinum (Pt) wire as a counter electrode (CE), a reference electrode (RE) of silver/silver chloride Ag/AgCl and PANI/Au NPs modified ITO electrode as a working electrode (WE). The three electrodes were connected to a potentiostat. Preparation of PANI/Au NPs modified ITO electrodes was done as was described in our earlier study^28^. SEM (JOEL-JSM-5400LV) was used to investigate the morphology of the Au NPs modified ITO and PANI/Au NPs modified ITO electrodes.
- Disposable SPE (SPE C110, DropSens, Spain), which consisted of carbon as working (4 mm diameter) and counter electrodes, and silver as a RE. SPE was connected to potentiostat by a connector (DSC, DropSens, Spain).

The electrochemical detection of *P*. *aeruginosa* was done by adding 10 ml of the overnight LB broth culture of the sample in the electrochemical cell when using PANI/Au NPs modified ITO electrode. Also, 10 µl of overnight LB broth culture of the sample was pipetted into the detector well of the SPE. The surface of the SPE was first subjected to chemically pretreatment based on immersing the SPE in a solution of NaOH (3 M) for 1 h, then rinsed with DIW and finally dried at 120 °C.

The electrochemical measurement was done using both the cyclic voltammetry (CV) scans (at a potential extending from −0.4 V to 0.0 V and scan rate 0.05 V/s) and the square wave voltammetry (SWV) scans were performed at frequency = 15 Hz, pulse amplitude 0.05 V, step potential = 0.004 V, initial potential = −0.7 V and final potential = 0.0 V.

### Isolation and identification of the bacterial isolates by the conventional methods

After the overnight culture in LB broth, all samples were sub-cultured on several selective culture media and incubated at 37 °C for 24 h. The *P*. *aeruginosa* isolates were presumptively recognized by the routine tests, including colony morphology, pigment production, and biochemical tests. Suspected colonies of *P*. *aeruginosa* on nutrient agar (large, opaque, irregular colonies with blue-green pigment), blood agar (smooth, large, flat colonies, pigment diffuses in the medium giving dark greenish-blue color and may produce diffuse hemolysis) and on MacConkey’s agar (pale showing non-lactose fermenter colonies) were further identified as Gram-negative, non-spore forming rods^31^ and oxidase-positive; showing deep purple color within 5–10 seconds of a single colony transfer onto oxidase disc (HiMedia Laboratories Ltd., India)^32^. *P*. *aeruginosa* also showed alkaline butt (red butt) and alkaline slant (red slant) when stabbed onto Triple sugar iron (TSI) (HiMedia Laboratories Ltd., India). *P*. *aeruginosa* isolates were citrate positive; changing the color of Simmon’s Citrate to the blue^33^. If no pigment was present after incubation for 48 h, further tests were used to identify the isolate completely^34^.

### Antimicrobial susceptibility testing

The antimicrobial susceptibility of the isolated *P*. *aeruginosa* isolates was done by the Kirby-Bauer disc diffusion method^35^, according to the CLSI guidelines, 2018^36^. The following antimicrobial discs were used (Hi-Media Laboratories Ltd., India): Amikacin (30 µg), Piperacillin (100 µg), Ceftriaxone (30 µg), Ciprofloxacin (5 µg), Cefoperazone-Sulbactam (25 µg), Imipenem (10 µg) and Meropenem (10 µg).

### Automated identification of the *P*. *aeruginosa* isolates

Pure *Pseudomonas* isolates were further characterized by the automated ID & Ast system for microbial identification and antibiotic susceptibility testing, MA120 (Render group, China), compliant with the manufacturer instructions.

### PCR amplification of the 16s rRNA gene of *P*. *aeruginosa* isolated strains

DNA was extracted by the boiling method^37^. The primer set used to amplify the 16 s rRNA gene of *P*. *aeruginosa* was as follows: Forward (5′GGGGGATCTTCGGACCTCA3′) and reverse (5′TCCTTAGAGTGCCCACCCG3′)^38^. PCR was performed using the SimpliAmp thermal cycler (Applied Biosystems, USA). The amplification procedure was done, as was previously described^38^. Briefly, initial denaturation was for 2 minutes at 95 °C. Then 25 cycles were completed, each consisting of denaturation at 94 °C for 30 s, annealing at 58 °C for 30 s, and extension at 72 °C for 50 s. Subsequently, a final extension of 5 minutes at 72 °C was applied. PCR products were resolved on 2% agarose gels with ethidium bromide and imaged with the EZ imager (Bio-Rad), where a band was visualized at the expected size of 956 base pair.

### Ethical approval

Written informed consent for the sample and data collection was obtained from each patient. It is worth to advise that all patients included in the study were able to sign the written informed consent by themselves because there was no single case that suffered from corneal ulcers in both eyes. Moreover, some patients experienced blurry vision but only in one eye. The local Ethical Committee of the Faculty of Medicine Assiut University appraised and accepted the study protocol in accordance with the latest revision of the Declaration of Helsinki (IRB NO. 17300293).

## Results

### Electrochemical detection of *P*. *aeruginosa* in corneal ulcer samples

**Figure 1:**
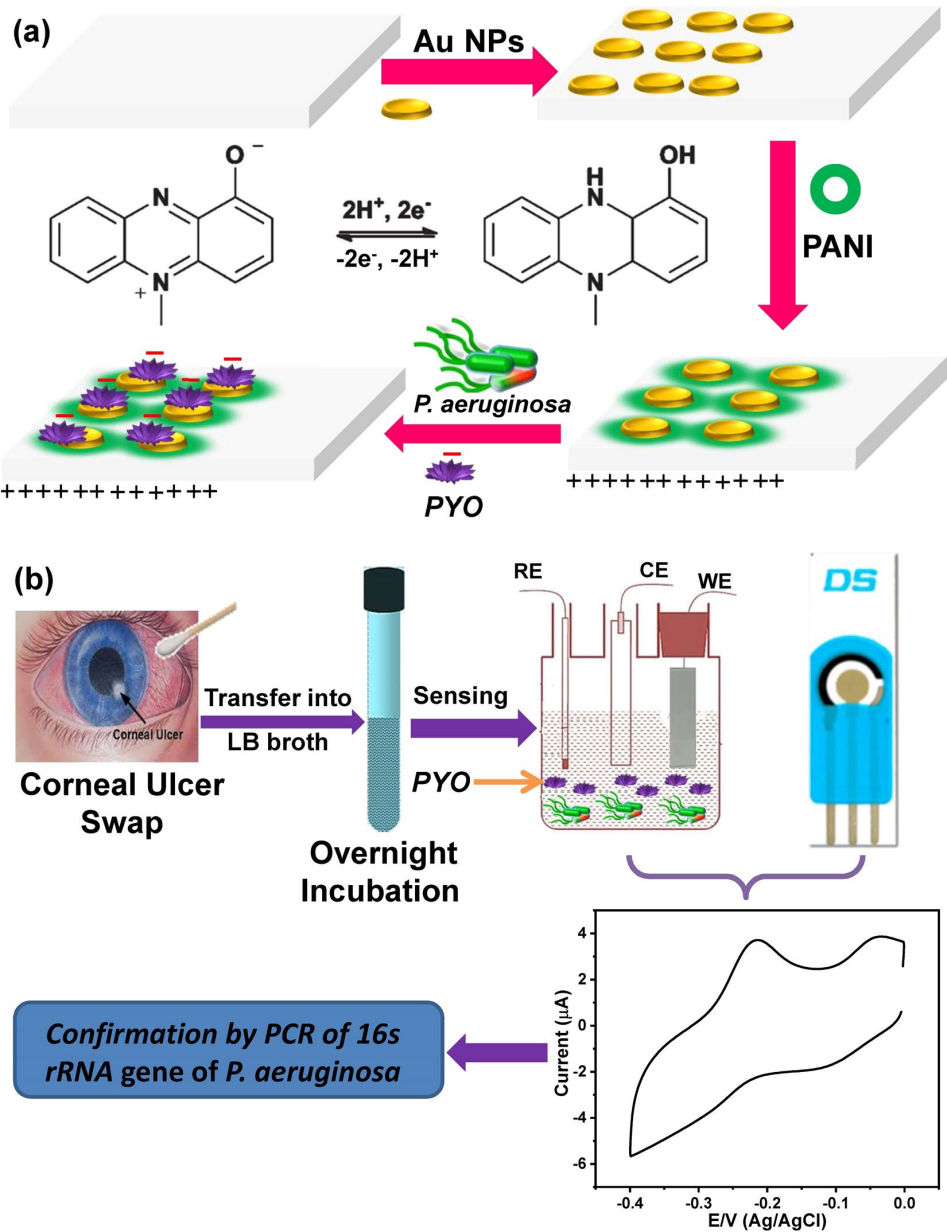
(**a**) The schematic diagram for fabrication of PANI/Au NPs/ITO electrode and the interaction between PANI and PYO and (**b**) the schematic diagram for electrochemical sensing of *P*. *aeruginosa* by using either PANI/Au NPs/ITO or SPE electrodes, the eye image was used form this website, https://www.pinterest.com/pin/7529524362981604/.

Here, PANI/Au NPs/ITO modified electrode with positive charges on the surface of the polymer layer was selected to monitor the negatively charged PYO molecules, and hence this electrostatic attraction force could enhance the rate of the mass transfer, as represented in Fig. (1a). To investigate the capability of both PANI/Au NPs modified ITO and the SPE electrodes, we have collected 50 corneal ulcer samples and studied their electrochemical behaviors based on CV and SWV techniques, as revealed in Fig. (1b).

### Using PANI/Au NPs modified ITO electrode

**Figure 2:**
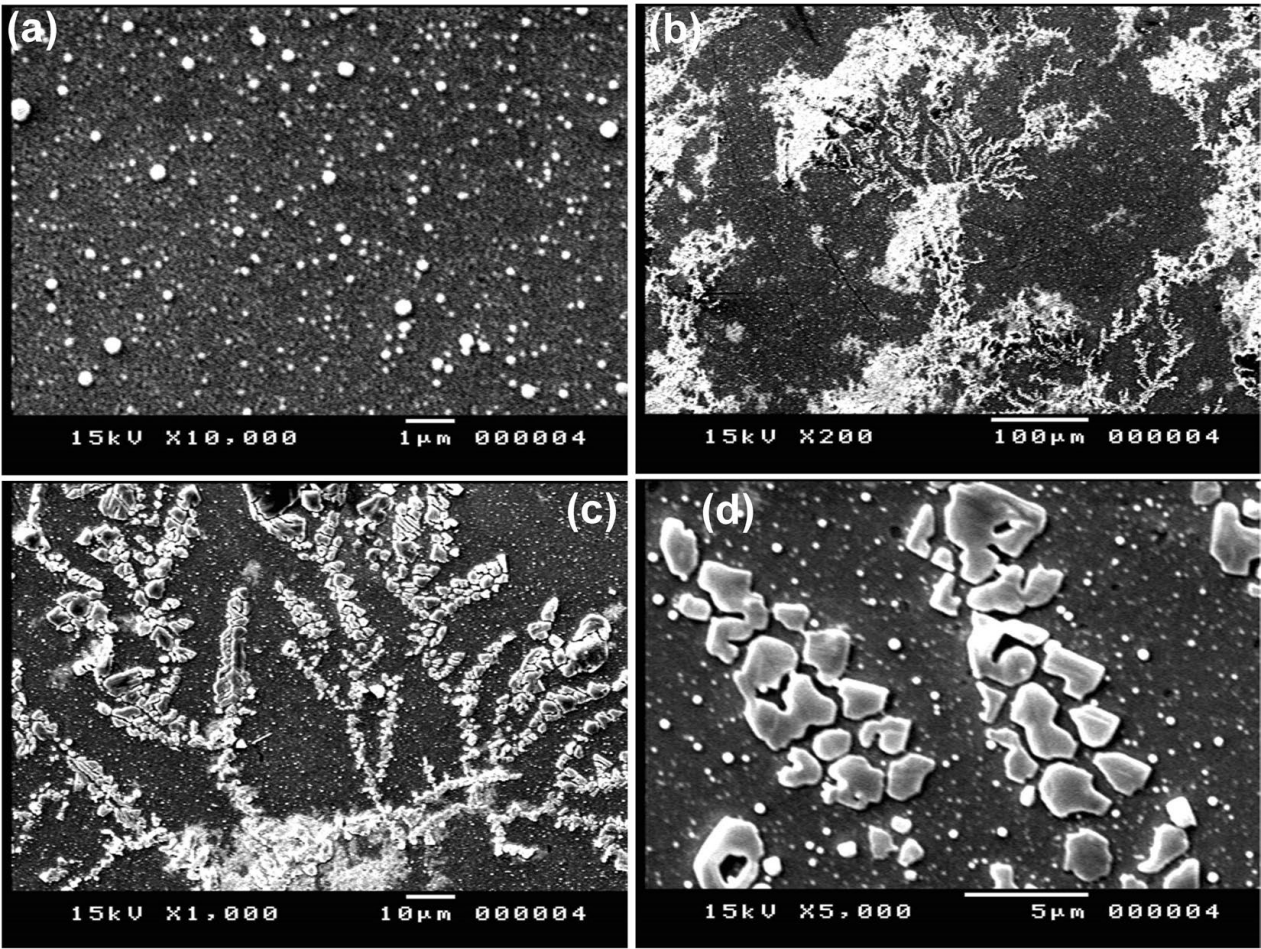
(**a**) SEM image of Au NPs modified ITO electrode and (**b**–**d**) SEM images of the PANI/Au NPs/ITO electrode.

**Figure 3:**
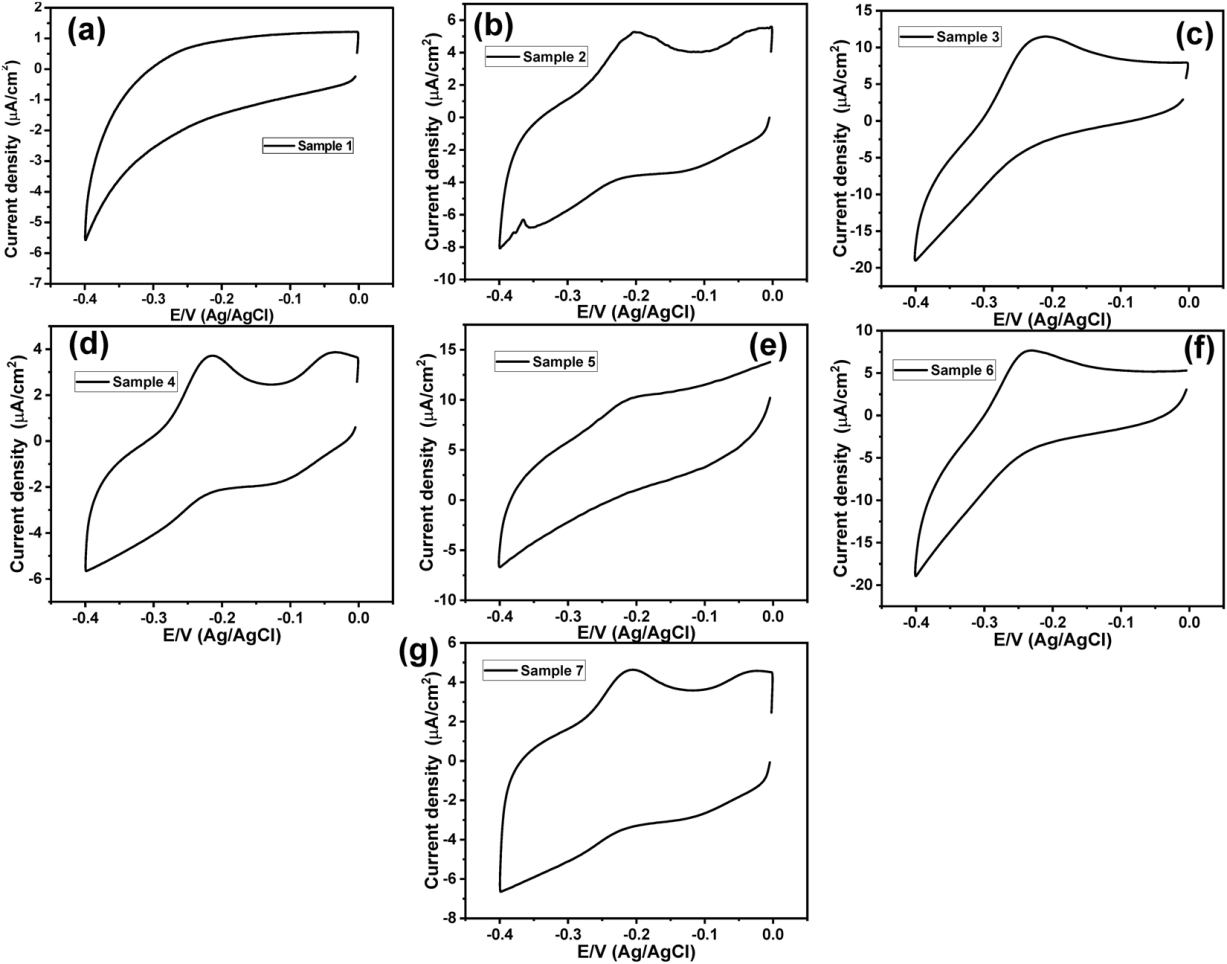
(**a**) Cyclic voltammogram of a negative sample and (**b**–**g**) Cyclic voltammograms of positive samples using PANI/Au NPs modified ITO electrode within a potential window (−0.4 V to 0.0 V), at a scan rate of 50 mV/sec.

The PANI/Au NPs modified ITO electrode was prepared according to our previous study^28^. Figure (2a) showed the SEM image of the Au NPs/ITO that indicated the formation of nanodots with an average size of about 95 nm. On the other hand, the morphology of the obtained PANI/Au NPs/ITO electrode was studied by using SEM images (Fig. (2b–d)), which confirmed the fabrication of a tree-like structure of PANI layer over the Au NPs. Figure (3a) represented a background CV response with no redox peaks detected within the PYO potential window (−0.4 V to 0.0 V). This behavior was obtained in 44 samples.

**Figure 4:**
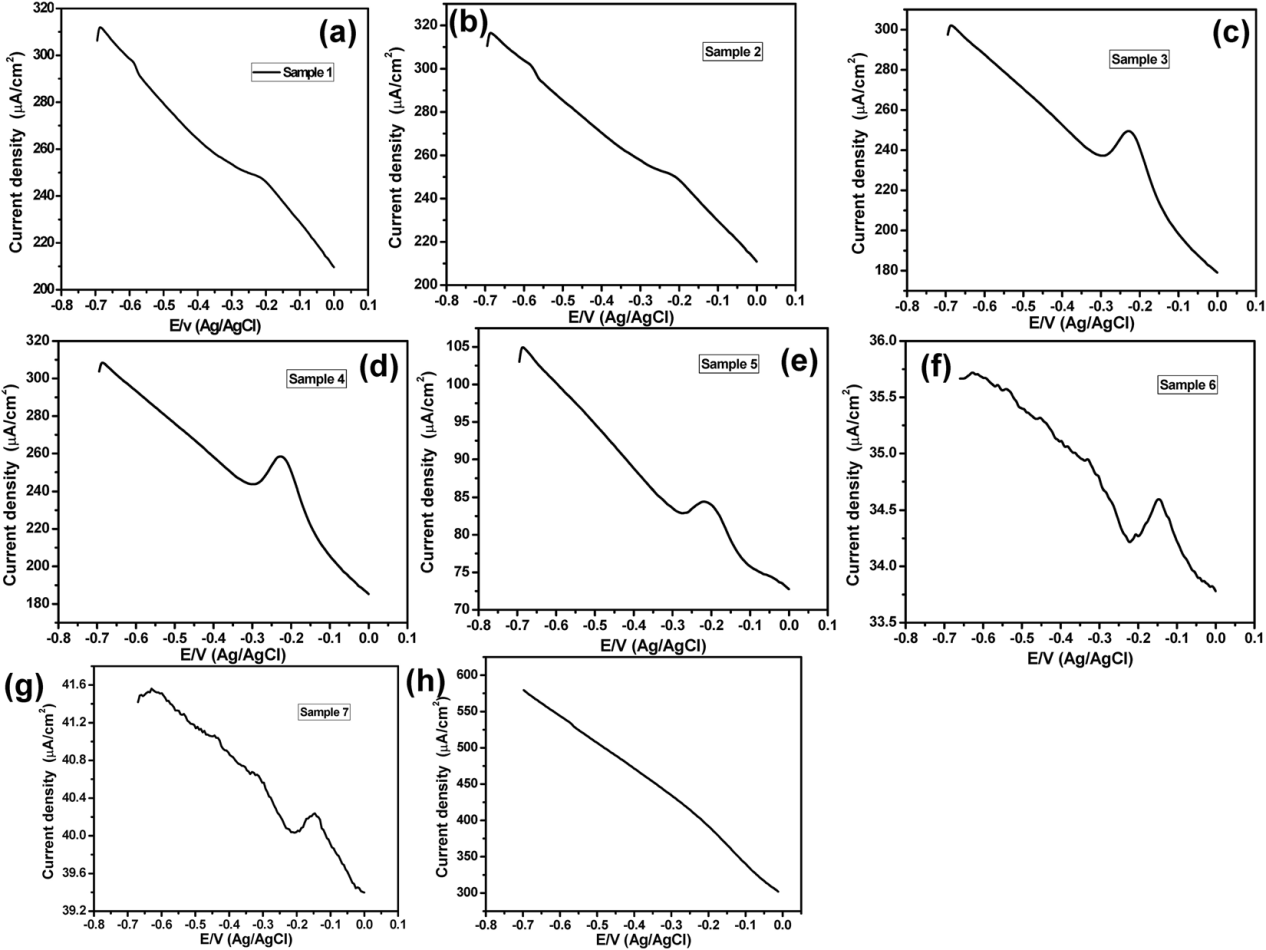
(**a**–**g**) SWV of positive samples, and (**h**) SWV of a negative sample, using PANI/Au NPs modified ITO electrode and in a potential window (−0.7 V to 0.0 V).

On the other hand, Fig. (3b–g) showed positive CV responses of positive samples, which represented an oxidation peak at about −0.23 V with different current intensities (a specific and unique peak of PYO). This was obtained only in 6 samples. To get more accurate and sensitive results we have applied the SWV technique instead of the CV technique. Figure (4a–g) represented the SWV responses from −0.7 V to 0.0 V of positive samples that showed an oxidation peak at −0.23 V. While Fig. (4h) showed the SWV response with no detectable redox peak within the PYO potential window (−0.7 V to 0.0 V) that distinguishes the negative sample.

The electrochemical results obtained by using the PANI/Au NPs modified ITO electrode were summarized in Table (1), which indicated that among the 50 tested corneal ulcer samples, 7/50 samples (14%) were electrochemically positive using SWV technique, while 6/50 samples (12%) were electrochemically positive using CV technique. The remaining samples were electrochemically negative.

**Table 1:** Comparison between the electrochemical detection results of *P*. *Aeruginosa* positive corneal ulcer samples using the PANI/Au NPs modified ITO electrode and the screen-printed electrode (SPE).

Sample number	PANI/Au NPs modified ITO		SPE	
	CV	SWV	CV	SWV
1	−ve	+ve	−ve	+ve
2	+ve	+ve	−ve	−ve
3	+ve	+ve	−ve	+ve
4	+ve	+ve	+ve	+ve
5	+ve	+ve	−ve	−ve
6	+ve	+ve	+ve	+ve
7	+ve	+ve	+ve	+ve

### Using the SPE sensor

**Figure 5:**
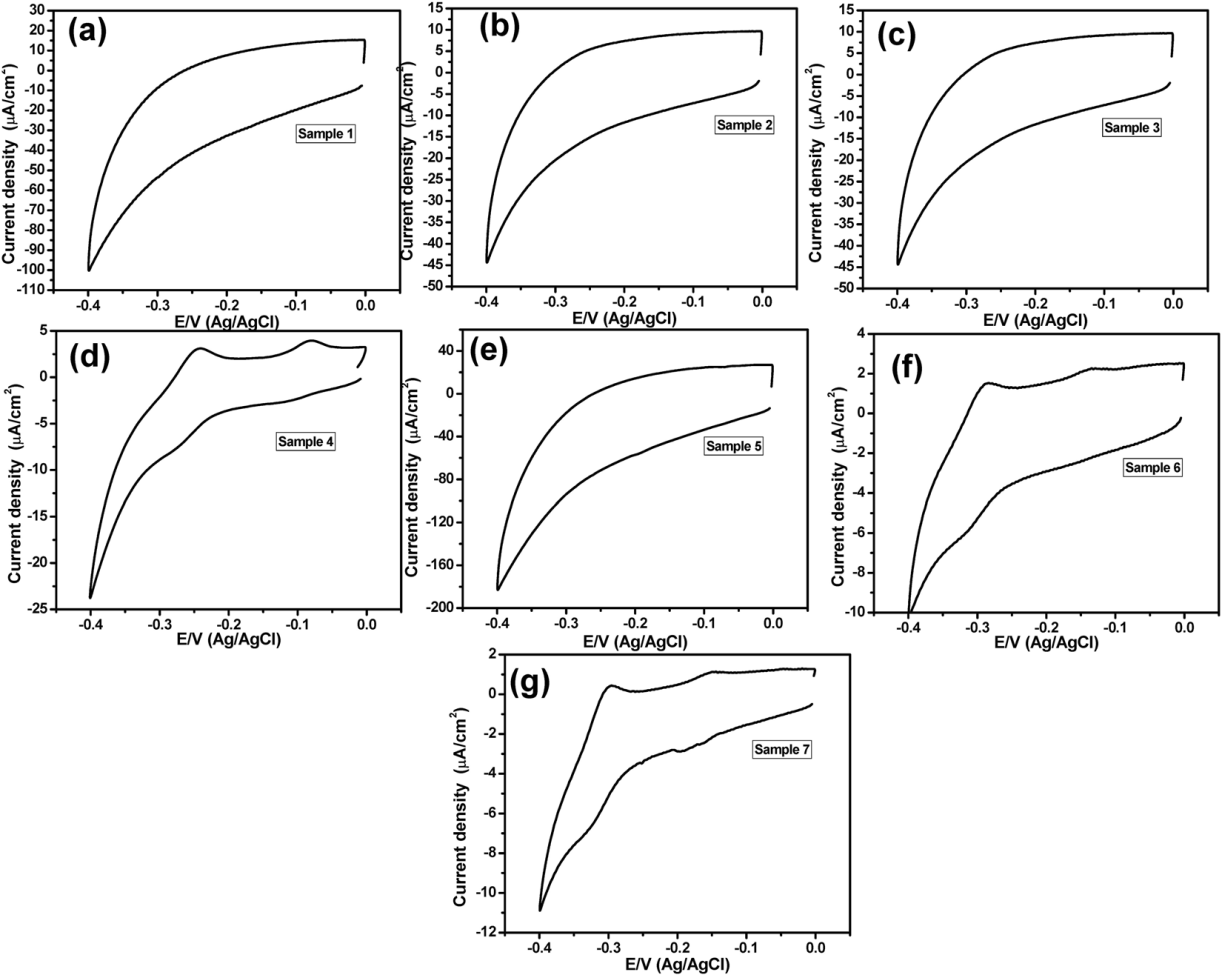
Cyclic voltammograms of (**a**–**c**,**e**) negative samples, and (**d**,**f**,**g**) positive samples, using SPE sensor within a potential window (−0.4 V to 0.0 V).

To validate the efficiency of the PANI/Au NPs modified ITO electrode for monitoring the PYO in corneal ulcer samples, SPE was used as a traditional electrode for detecting the PYO in the 50 samples instead of PANI/Au NPs modified ITO electrode under the same conditions. Figure (5a–c,e) showed the CV responses of negative samples within the PYO potential window (−0.4 V to 0.0 V) that didn’t show any redox peak. Furthermore, the CV responses of positive samples using SPE sensor that represented an oxidation peak at about −0.30 V were shown in Fig. (5d,f,g).

**Figure 6:**
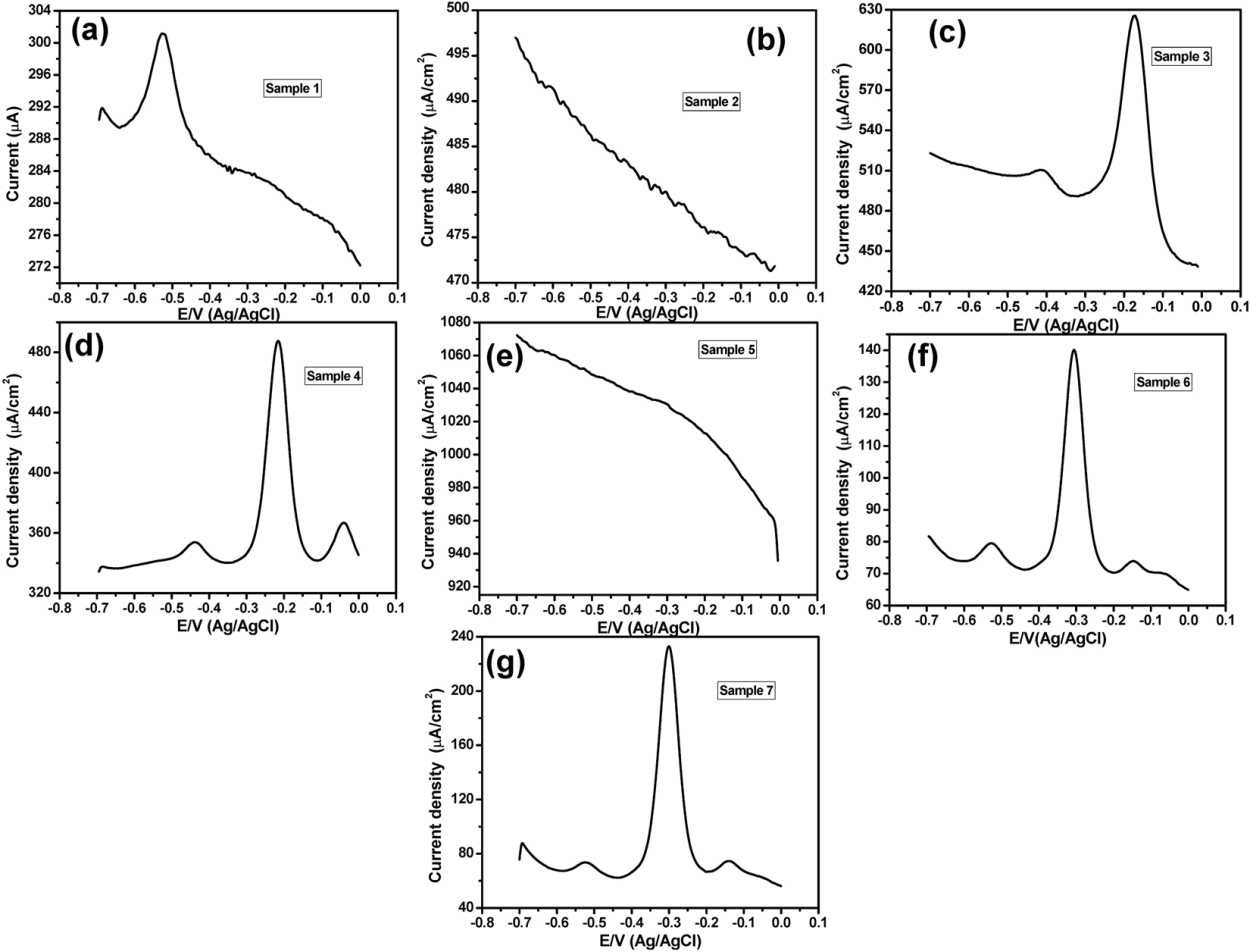
(**a,c,d,f,g**) The square wave voltammograms of positive samples, and (**b**,**e**) the SWV responses of negative samples, using the SPE sensor within a potential window (−0.7 V to 0.0 V).

Moreover, the SWV technique was also used to study the performance of SPE in detecting PYO. For 45 samples, no oxidation peaks could be observed within the potential range from −0.7 V to 0.0 V as shown in Fig. (6b,e) which means these 45 samples are negative samples. Furthermore, the SWV responses of four samples (samples# 3, 4, 6 and 7) have shown a set of oxidation peaks including the characteristic oxidation peak of PYO molecules at about −0.22 V **(**Fig. 6c,d,f,g) and hence theses samples are positive samples. On the other hand, the SWV of the sample# 1 showed a strong oxidation peak at about −0.52 V in addition to a very weak and broad peak at about −0.25 (Fig. 6a), so this sample could be considered as a positive sample. The above data were tabled in comparison with the PANI/Au NPs modified ITO electrode in Table 1. Among the 50 corneal ulcer samples, 5/50 samples (10%) were electrochemically positive using the SWV technique, while 3/50 samples (6%) were electrochemically positive using the CV technique. It is worth to note that although the SW voltammograms of the SPE positive samples have shown sharper oxidation current peaks in comparison with those observed at the PANI/Au NPs/ITO electrode, the oxidation peak was detectable only in five samples in case of using the SPE while the PANI/Au NPs/ITO electrode detected easily the oxidation peak in 7 samples. Thus, the PANI/Au NPs/ITO electrode is more accurate and selective for PYO detection.

**Figure 7:**
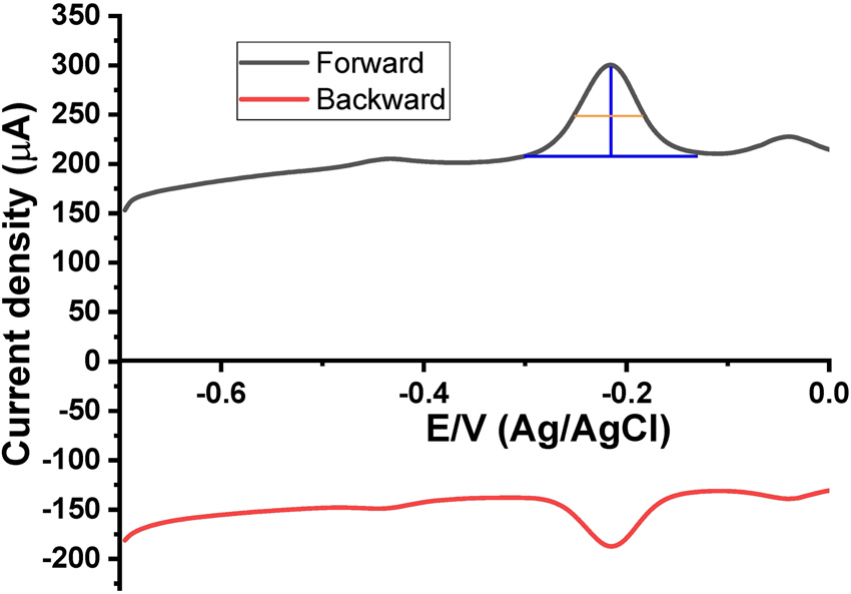
The forward and backward SW voltammograms of PYO.

In order to study the kinetics of the electrode reaction, we have measured the half-peak width of the SW voltammograms based on a previously reported method^39^. Figure 7 showed the forward and backward SW voltammograms of PYO at a frequency (*f)* of 15 Hz, amplitude (*E*_sw_) = 0.05 V, and potential increment (Δ*E*) = 0.004 V. From Fig. 7 we found that the peak potential (Δ*E*_p_) = −216 mV, peak current (Ψ_p_) = 301 µA and the half-peak width (ΔEp/2) = 65 mV.

The dimensionless electrode kinetic parameter (K) was calculated from Eq. (1).

1 $$\Delta \text{Ep}/2=-\,10.85\,\mathrm{ln}({\rm{K}})+80$$

So, ln(K) = −25.85, and hence K = 3.25

The standard rate constant of electron transfer (*k*_s_) was calculated from Eq. (2).

2 $${\rm{K}}={k}_{s}{({\rm{fD}})}^{-0.5}$$

where, D (cm^2^.s^−1^) is the diffusion coefficient.

So, ks = 0.00315 cm.s^−1^

### Isolation and phenotypic identification of *Pseudomonas aeruginosa* by conventional bacteriologic identification methods

All collected samples were investigated by using the conventional bacteriologic identification methods and the results were summarized in Table 2. Of the 50 corneal ulcer samples, 30/50 (60%) were due to bacterial infections, while the other 20/50 (40%) cases were negative for bacterial growth. Of the bacterial isolates, 14/30 (46.7%) were *Staphylococcus spp*. [11/30 (36.7%) were coagulase-positive while 3/30 (10%) were coagulase-negative], 2/30 (6.7%) were *Streptococci*. On the other hand, 9/30 (30%) were *Pseudomonas spp*. [5/30 (16.7%) were *P*. *aeruginosa* while 4/30 (13.3%) were non*-aeruginosa*] and 5/30 (16.7%) were *Klebsiella*. All the seven *P*. *aeruginosa* isolates were resistant to ceftriaxone, four were resistant to ciprofloxacin, three resistant to imipenem, meropenem, cefoperazone-sulbactam and piperacillin and two were resistant to amikacin.

**Table 2:** Phenotypic identification of *P*. *aeruginosa* in the corneal ulcer samples.

Bacterial pathogens	# of isolates	Percentage of isolates
*Staphylococcus spp*.	14	46.7 (%)
*Coagulase* +*ve*	11	36.7 (%)
*Coagulase −ve*	3	10 (%)
*Streptococcus pneumoniae*	2	6.7 (%)
*Pseudomonas spp*.	9	30 (%)
*P*. *Aeruginosa*	5	16.7 (%)
*Non Aeruginosa*	4	13.3 (%)
*Klebsiella*	5	16.7 (%)

### Automated identification of the *P*. *aeruginosa* isolates

Six of the nine *Pseudomonas* spp. isolates were of the *aeruginosa* spp., while 3/9 where *P*. *fluorescence* by the automated ID & Ast system.

### PCR amplification of the 16 s rRNA gene of *P*. *aeruginosa*

All *Pseudomonas* spp. isolates were tested by PCR. Figures S1–S3 showed the gel electrophoresis of the PCR-amplified products for the detection of the 16 s rRNA gene for nine samples, which indicated that 7/9 isolates were having the 16 s rRNA gene of *P*. *aeruginosa*.

### Sensitivity and specificity of the electrochemical, conventional and automated methods compared with PCR amplification of 16 s rRNA gene for the detection of *P*. *aeruginosa* in corneal ulcer samples

A summary of the results of the different methods for the identification of *P*. *aeruginosa* was illustrated in Table (3). All *P*. *aeruginosa* isolates identified by PCR (100%) were also positive by the SWV using PANI/Au NPs modified ITO. Meanwhile, 6/7 (85.7%) previous isolates were positive by both the CV using PANI/Au NPs modified ITO and the automated ID & Ast system. Only 5/7 (71.4%) isolates were positive by both conventional methods and SWV using SPE. CV using SPE could only detect 3/7 isolates (42.9%) of *P*. *aeruginosa*.

**Table 3:** Summary of the results of *P*. *aeruginosa* detection by electrochemical, phenotypic, automated and *16* *s rRNA* gene PCR methods.

Detection method	Number of positive samples	Percentage
PCR amplification of 16 s rRNA gene	7	**100%**
SWV using PANI/Au NPs modified ITO	7	**100%**
CV using PANI/Au NPs modified ITO	6	85.7%
The automated ID & Ast system	6	85.7%
SWV using SPE	5	71.4%
Phenotypic methods	5	71.4%
CV using SPE	3	42.9%

CV cyclic voltammetry scans, SWV square wave voltammetry scans, SPE screen-printed electrode.

Percentages are calculated from *P*. *Aeruginosa* positive samples by PCR amplification of 16s rRNA gene.

Sensitivities and specificities of the electrochemical methods and conventional methods for the detection of *P*. *aeruginosa* in corneal ulcer samples compared with PCR amplification of the 16 s rRNA gene of *P*. *aeruginosa* were reported in Table (4). SWV using PANI/Au NPs modified ITO has shown 100% sensitivity, specificity, positive and negative predictive values. The remaining methods have only revealed 100% specificities and positive predictive values. The CV using PANI/Au NPs modified ITO and the automated ID & Ast system have shown better sensitivities (85.7%) than both the SWV using SPE and the conventional identification methods (71.4%) followed by the CV using SPE (42.9%). Similarly, higher negative predictive values were obtained by the CV using PANI/Au NPs modified ITO and the automated ID & Ast system (66.6%) followed by both SWV using SPE and the phenotypic identification methods (50%) then by the CV using SPE (33.3%).

**Table 4:** Sensitivities and specificities of the electrochemical, phenotypic and automated identification methods of *P*. *aeruginosa* compared with PCR amplification of the *16s rRNA* gene.

	PANI/Au NPs/ITO electrode		Screen printed electrode		Phenotypic methods	Automated Method
	CV	SWV	CV	SWV		
Sensitivity	85.7%	**100%**	42.9%	71.4%	71.4%	85.7%
Specificity	100%	**100%**	100%	100%	100%	100%
PPV	100%	**100%**	100%	100%	100%	100%
NPV	66.6%	**100%**	33.3%	50%	50%	66.6%

CV cyclic voltammetry scans, SWV square wave voltammetric scans, PPV positive predictive value, NPV negative predictive value.

## Discussion

The bacterial corneal disease remains the leading cause of blindness globally, and it often occurs following trauma or eye injury. Typically corneal ulcer is characterized by red-eye, mild to severe ocular discharges, pain, and reduced vision^40^. Pascolini and Mariotti, 2012 reported that corneal opacities, caused by an infectious corneal ulcer, are the fourth leading cause of blindness worldwide and represent 10% of visual impairment, particularly in developed countries^41^. In the United States, infectious keratitis is often associated with contact lens wear^42^; however, in developing countries, it is commonly associated with ocular trauma occurring during farming^43^. In India, approximately 2 million people develop a corneal ulcer every year^44^. Unfortunately, corneal damage can occur within 24 h, especially with the serious infections caused by highly virulent opportunistic pathogens, frequently as *P*. *aeruginosa*^45^.

The ubiquitous gram-negative bacteria *P*. *aeruginosa* is multidrug-resistant and frequently identified in biofilms^46^. It causes severe infections in immune-compromised patients^47^ that are difficult to eradicate with antibiotics^48^. Delays in obtaining results of the conventional identification methods influence both patient care, and the ability to decide an appropriate antibiotic therapy. Moreover, in spite of the improvements in the rapid molecular techniques, the use of cultures remains the standard practice in laboratories^48^. These days, there is an urgent need to find rapid and precise approaches for bacterial identification because the culture procedure can take from 24 to 48 h to get results. Early diagnosis of *P*. *aeruginosa* is a highly required goal in hospitals that consequently will improve patients care outcomes.

PYO is a redox-active molecule produced by *P*. *aeruginosa*^49^ and is considered an excellent biomarker for *P*. *aeruginosa* that could be used as a selective alternative for direct detection. Great efforts were made to expand the applications of the electrochemical technologies in health, environment, and security. A variety of printed sensors and biosensors have been developed within the last few years^50–52^, to improve the routine diagnostic tools. Preceding studies have described the electrochemical detection of PYO in *P*. *aeruginosa* identification^5,10,30,53–55^. Still, none of the previous electrochemical detection methods have been used to identify *P*. *aeruginosa* in corneal ulcers, and none of them tested the use of the ITO electrode as a working electrode for the detection of PYO.

In our preliminary work^28^, we have established the design and operation of PANI/Au NPs/ITO electrode which has shown a fast, 100% sensitivity and specificity and excellent selectivity for PYO at extremely low concentrations in the presence of interfering substances like ascorbic acid, uric acid, and glucose. We used the scanning electron microscope and CV to characterize the fabricated electrode. The detection limit of PYO was 500 nM, with a linear detection range from 238 µM to 1.9 µM. The modified electrode had a four-fold enhanced performance compared with the published results of the SPE.

In this study, we evaluated the efficiency of the modified ITO sensor on the detection of *P*. *aeruginosa* in 50 corneal ulcer samples and compared the results with the SPE sensor, conventional and automated identification methods using the PCR amplification of 16 s rRNA gene as a gold standard test for *P*. *aeruginosa* identification. *P*. *aeruginosa* was the most commonly isolated gram-negative bacteria in this study. We have found that 7/50 samples were positive by PCR and SWV using PANI/Au NPs modified ITO electrode. While six of those seven samples (85.7%) were positive by the automated system and by the CV using PANI/Au NPs modified ITO electrode, 5/7 (71.4%) samples were positive by the conventional phenotypic methods and by SWV using SPE and 3/7 (42.9%) by the CV using SPE. All positive samples had cyclic voltammograms with a unique oxidation peak of PYO at −0.23 V when using PANI/Au NPs modified ITO electrode while −0.30 V when using SPE. This small potential shift in the location of the PYO peak may be attributed to the limited stability of the silver reference electrode of the disposable SPE sensor^5^.

Isolation of pathogens is still done by the conventional culture methods in a large number of laboratories because it is convenient and manageable. Nevertheless, culture media and biochemical tests are not entirely specific and sometimes fail to offer probable identification, especially up to the species level. Therefore, additional verification of species identity is frequently needed, which is time and money consuming^56^. Two of our corneal ulcer samples were not considered *P*. *aeruginosa* by the phenotypic identification methods as they did not produce a visible pigment on the culture media. Meanwhile, these two samples were positive by PCR amplification of the 16 s rRNA gene of *P*. *aeruginosa*. It was recorded earlier that not all strains of *P*. *aeruginosa* can produce pigment on the culture medium^34^. These strains may have produced PYO, but its amount was too small to be seen on the culture medium, but the highly sensitive SWV technique using PANI/Au NPs/ITO electrode was capable of detecting these limited amounts of PYO.

Different non-culture based approaches are currently used to provide rapid bacterial identification and antibiotic susceptibility simultaneously^56^. But in these methods, it is impossible to process directly patient samples^57^, and there is a great need for a pure culture of the bacteria and long processing time^56^. Despite that, in our study, the automated system has shown better sensitivity in detecting *Pseudomonas* spp. than the conventional methods; one case was misinterpreted for *P*. *fluorescence* instead of *P*. *aeruoginosa*.

As far as we know, it is the first study to evaluate the use of electrochemical sensors in the detection of *P*. *aeruginosa* in corneal ulcers. We have demonstrated that the electrochemical detection of PYO by the SWV technique using PANI/Au NPs modified ITO electrode was the only technique showing 100% agreement with the molecular method in sensitivity, specificity, positive and negative predictive values compared with the SPE, conventional and automated methods. The modified electrode was able to discriminate between *P*. *aeruginosa*, *Staphylococcus spp*., *Streptococci*, and *Klebsiella*. This endorses our previous findings^28^ that using the modified electrode allows fast, accurate, and selective detection of *P*. *aeruginosa* in mixed bacterial cultures without the need for isolation of pure colonies of bacteria and in the presence of interfering molecules.

The result obtained by the modified sensor was better than that of the disposable electrochemical sensor used by Sismaet and his collaborators^54^ for rapid screening for *P*. *aeruginosa* in fluids collected from chronic wounds, which demonstrated a sensitivity of 71% and specificity of 57%^58^. SWV technique showed higher sensitivity to PYO than CV technique. These results were attributed to the drawbacks of the CV, which introduce a huge capacitive background current (or noise). This noise makes it difficult to recognize the faradaic current, which is the desired signal and rigorously disturbs the signal to noise ratio. Since SWV is a pulsed technique, it can discriminate against the charging current and eliminate this drawback. For this reason, the SWV technique can detect a very low concentration of PYO in the clinical sample.

In summary, we have studied the electrochemical capability of the developed electrochemical sensor for the detection of PYO by SWV technique in 50 samples collected from patients suffering from corneal ulcers. The obtained results indicated that the electrochemical detection of PYO by SWV technique using PANI/Au NPs modified ITO electrode was the only technique showing 100% agreement with the molecular method in sensitivity, specificity, positive and negative predictive values when compared with the SPE, conventional and automated methods (phenotypic and 16* s rRNA* gene PCR methods). Therefore, PANI/Au NPs modified ITO electrode is recommended as a fast, cheap, accurate, and selective PYO biomarker sensor based on the SWV technique for the detection of *P*. *aeruginosa* in cases of corneal ulcer.

## Supplementary information

Supplementary information
