# Supplementary material for: Highly specific Electrochemical Sensing of Pseudomonas aeruginosa in patients suffering from corneal ulcers: A comparative study
Source: Sci Rep. 2019 Dec 4;9:18320. doi: 10.1038/s41598-019-54667-0 (PMC6892848; doi:10.1038/s41598-019-54667-0)
Supplement: Supplementary file 1 — Supplementary information [file 41598_2019_54667_MOESM1_ESM.docx]

**Highly specific Electrochemical Sensing of *Pseudomonas aeruginosa* in patients suffering from corneal ulcers: A comparative study**

**Marwa M. Khalifa ^1^,  Amal A. Elkhawaga ^2^, Mona A. Hassan^2^, Asmaa M Zahran^3^, Ahmed M Fathalla^4^, Waleed A. El-Said^5,6^**^*^**, Omnia El-Badawy^2^**

# Khaled Saad

# Search for articles by this author

#### Affiliations

# Children’s Hospital, Assiut University, Egypt

*^1^Department of Microbiology and Immunology, Faculty of Pharmacy, Assiut University, Assiut, 71526, Egypt*

*^2^Department of Medical Microbiology and Immunology, Faculty of Medicine, Assiut University, Assiut, 71515, Egypt*

*^3^Department of Clinical Pathology, South Egypt Cancer Institute, Assiut University, Assiut, Egypt ^4^Department of Ophthalmology, Faculty of Medicine, Assiut University, Assiut, 71515, Egypt*

*^5^Department of Chemistry, Faculty of Science, Assiut University, Assiut, 71516, Egypt*

*^6^Chemistry Department, Faculty of Science, University of Jeddah, P.O. 80327, Jeddah 21589, Saudi Arabia*

* Correspondence to:

Waleed A. El-Said

Department of Chemistry, Assiut University, Egypt

Tel. +2-088-2412405; Fax: +2-088-2342708

E-mail address: [awaleedahmed@yahoo.com](mailto:awaleedahmed@yahoo.com); [waleed@aun.edu.eg](mailto:waleed@aun.edu.eg)

Orcid ID [/0000-0002-7052-7612](https://orcid.org/0000-0001-8445-711X)


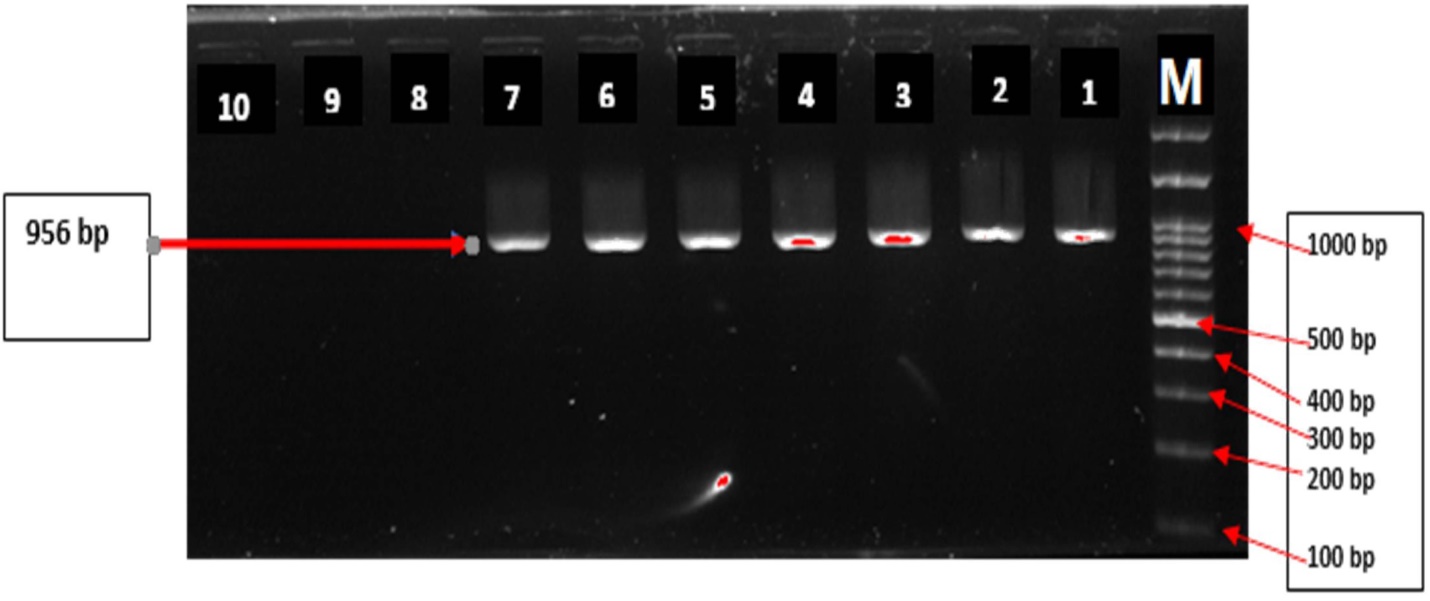


**Figure S1**. Gel electrophoresis of the PCR-amplified products for the detection of the 16s rRNA gene. Lane M is a DNA marker (100-1500 bp); lane 10 is a negative control and lanes from 1 to 7 are positive for the 16s rRNA gene (965bp). Lanes 8 and 9 are negative for the 16s rRNA gene.


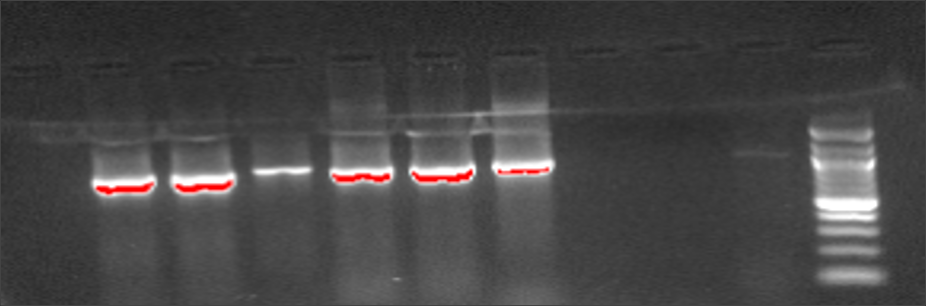


10 9 8 7 6 5 4 3 2 1 M

**Figure S2.** The gel electrophoresis of the PCR-amplified products for detection of *16s rRNA* gene of *P. aeruginosa*. Lane M is DNA marker (100-1500 bp). Lane 10 is a negative control. lanes 1 and from 4 to 9 are positive for the *16s rRNA* gene (965bp). Lanes 2 and 3 are negative for the*16s rRNA* gene.


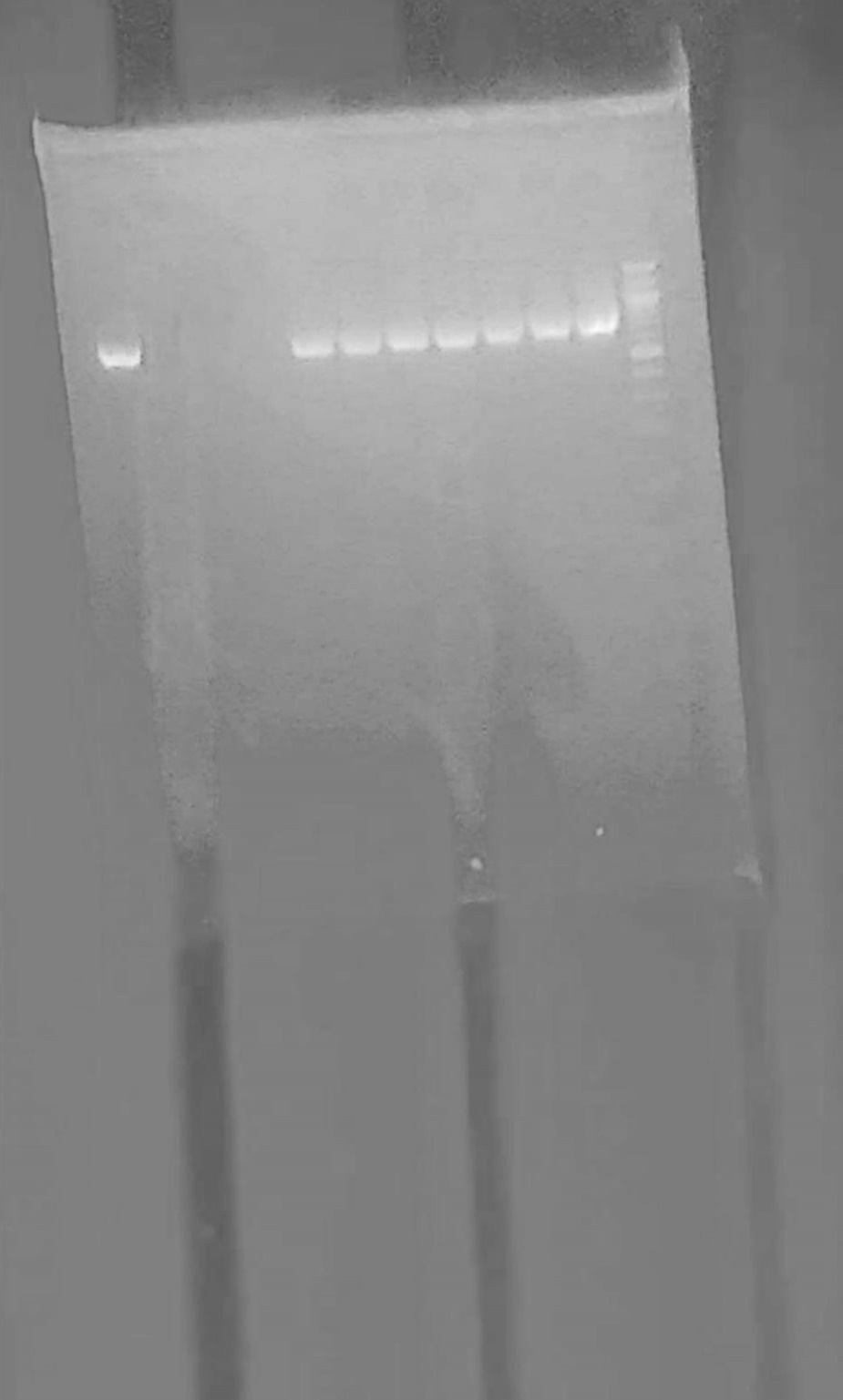


**Figure S3.** The full-length gel electrophoresis of the PCR-amplified products for detection of *16s rRNA* gene of *P. aeruginosa*.
